# PLGA–TiO_2_ as a Carrier System for Drug Release

**DOI:** 10.3390/ijms231810755

**Published:** 2022-09-15

**Authors:** M. I. Torres-Ramos, M. F. Martín-Marquez, María del Carmen Leal-Moya, Suresh Ghotekar, Jorge Alberto Sánchez-Burgos, Alejandro Pérez-Larios

**Affiliations:** 1 Laboratorio de Investigación en Nanomateriales, Agua y Energía, Departamento de Ingeniería, Centro Universitario de los Altos, Universidad de Guadalajara, Av. Rafael Casillas Aceves 1200, Tepatitlán de Morelos 47620, Jalisco, Mexico; 2 Especialidad en Endodoncia, Centro Universitario de los Altos, Universidad de Guadalajara, Av. Rafael Casillas Aceves 1200, Tepatitlán de Morelos 47620, Jalisco, Mexico; 3 Department of Chemistry, Smt. Devkiba Mohansinhji Chauhan College of Commerce and Science, University of Mumbai, Silvassa 396 230, Dadra and Nagar Haveli (UT), India; 4 Laboratorio de Investigación en Alimentos, Tecnologico de Tepic, Tecnologico Nacional de México. Av. Tecnologico 2595, Tepic 63175, Nayarit, Mexico

**Keywords:** TiO_2_ nanoparticles, PLGA, drug delivery, functionalization, biomaterials

## Abstract

This paper reports the results of the PLGA–TiO_2_ nanocomposite regarding the green synthesis of titanium dioxide nanoparticles using a natural extract, its characterization, and encapsulation with poly(lactic-co-glycolic acid) (PLGA). UV–visible spectrometry was used for the identification of terpenes present in the extracts. The morphology of the nanoparticles was determined by scanning electron microscopy. Infrared spectroscopy was used for the determination of functional groups, while X-ray diffraction was used to determine the crystal structure. The analysis of the extended release of the encapsulated extract in the matrix of the nanomaterial resulted in a maximum visible UV absorbance at approximately 260 nm and confirmed the synthesis of titanium dioxide nanoparticles. Moreover, terpenes enhance synthesis and stabilize titanium dioxide nanoparticles. The synthesized structures are spherical and amorphous, 44 nm in size, and encapsulated at 65 nm.

## 1. Introduction

Biomedical applications of nanomaterials have received considerable attention from researchers [1]. Titanium dioxide (TiO_2_) has been used mainly for water treatment specifically by advanced oxidation processes and as an antibacterial agent [2]. TiO_2_ consists of three phases: anatase, rutile, and brookite [3]. The anatase phase is the most active in photocatalysis [4,5,6]. Recently, we have sought to synthesize TiO_2_ nanoparticles that produce mesoporous spheres with hydrophobic properties, which can be used in medicine and pharmacology [7,8]. On the other hand, polymers have played an important role in conventional pharmaceutical formulations; these are used as pharmacological agents [9]. Among the most commonly used is poly(lactide-co-glycolide) (PLGA), mainly to control the release of the drug [10]. One of the ways to improve both the physical characteristics and the biological properties of nanomaterials is the development of polymer nanomaterials as nanocomposites, making use of biocompatible materials such as PLGA [11,12].

The incorporation of nanoparticles such as TiO_2_ within a polymer matrix improves its physical properties because the intertwining of the networks at the molecular level cannot be separated unless the chemical bonds are broken [13]. In addition, the morphology of this polymeric network generates a synergy with the initial components and allows for controlled releases [9]. TiO_2_ is low-cost and non-toxic [14] in addition to being approved by the US Food and Drug Administration (FDA) for the food industry and the medical field (except in the European territory) [15]. This places it as a nanomaterial used for biomedical applications, specifically for drug release [16].

In the present work, a nanocomposite (PLGA–TiO_2_) based on TiO_2_ nanoparticles (TiO_2_ NP) synthesized by green chemistry and functionalized with a natural extract, encapsulated with PLGA, is studied. Using scanning electron microscopy (SEM), infrared (FT-IR) and ultraviolet-visible spectroscopy (UV–Vis), physisorption analysis, and X-ray diffraction (XRD), the physicochemical properties of the nanocomposite material were characterized and the release time was studied.

## 2. Results and Discussion

### 2.1. SEM Analysis

**Figure 1:**
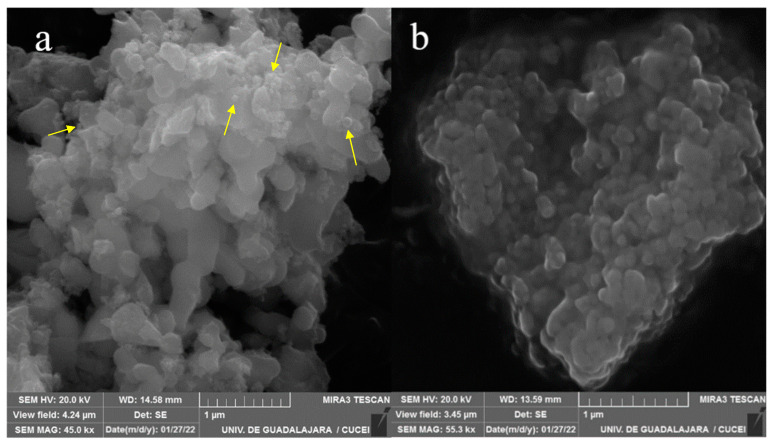
Micrographs of nanoparticles. ((**a**): TiO_2_ NP, (**b**): PLGA-TiO_2_).

The micrographs obtained by SEM demonstrate the morphology of the synthesized nanomaterial. Figure 1a, belonging to the TiO_2_ NPs, presents nano agglomerates and spherical morphologies (shown in the image) characteristic of TiO_2_ [12]. Using Image J software, it was determined that the spheres present in the sample have an approximate size of 44 nm. These spheres cluster together to form clusters that are encapsulated by the PLGA polymer matrix shown in Figure 1b. This polymer layer prevents us from seeing the exact morphology of the material it contains; however, it is observable that the nanocomposite was encapsulated.

### 2.2. XRD Analysis

**Figure 2:**
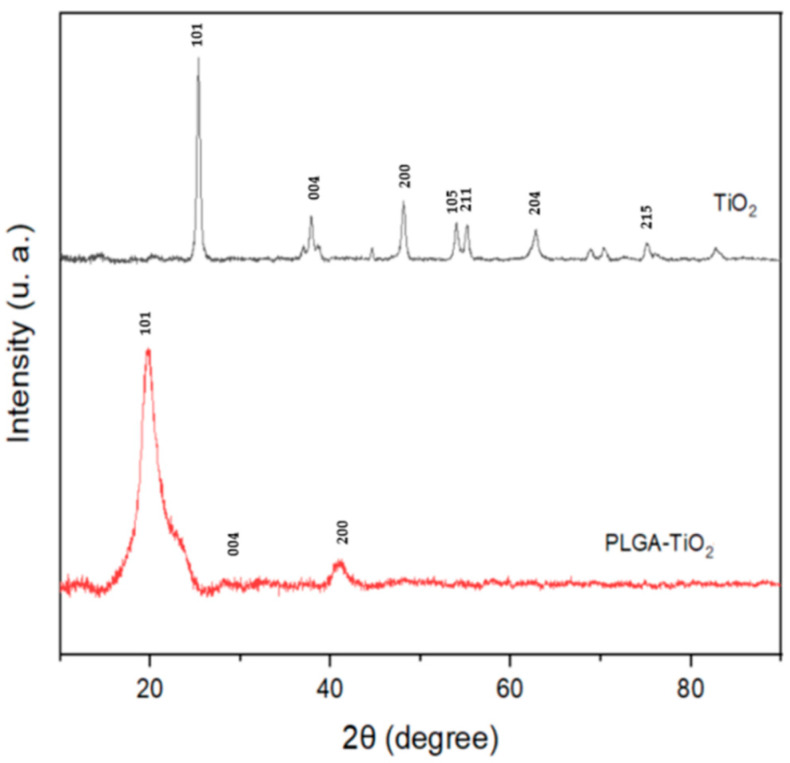
X-ray diffraction patterns of pure titanium dioxide (TiO_2_) and nanocomposite (PLGA–TiO_2_).

TiO_2_ in the anatase phase was selected as a material to work with due to its high surface area; having greater surface availability can help obtain a better functionalization of the nanoparticle [17]. XRD analysis was used to study the structure and phase formation of the sample. The diffraction spectrum (Figure 2) confirmed the presence of the anatase phase in our nanomaterial, showing the characteristic peaks of the phase (101, 004, 200) that are in agreement with the crystallographic chart JCPDS 21-1272 for titanium dioxide in the anatase phase. The crystallite size and lattice parameters (Table 1) were obtained using Scherrer’s equation and Bragg’s law, respectively, demonstrating that there are no differences between the parameters reported for TiO_2_ [4,12,18], meaning that green synthesis does not modify the crystallinity and morphology of the material. The diffraction pattern of the PLGA–TiO_2_ nanocomposite is mostly amorphous. However, peaks that are similar to those that correspond with the anatase phase of TiO_2_ are observed, which indicates the housing of the nanoparticles in the polymer matrix [19].

### 2.3. UV-Vis Analysis

**Figure 3:**
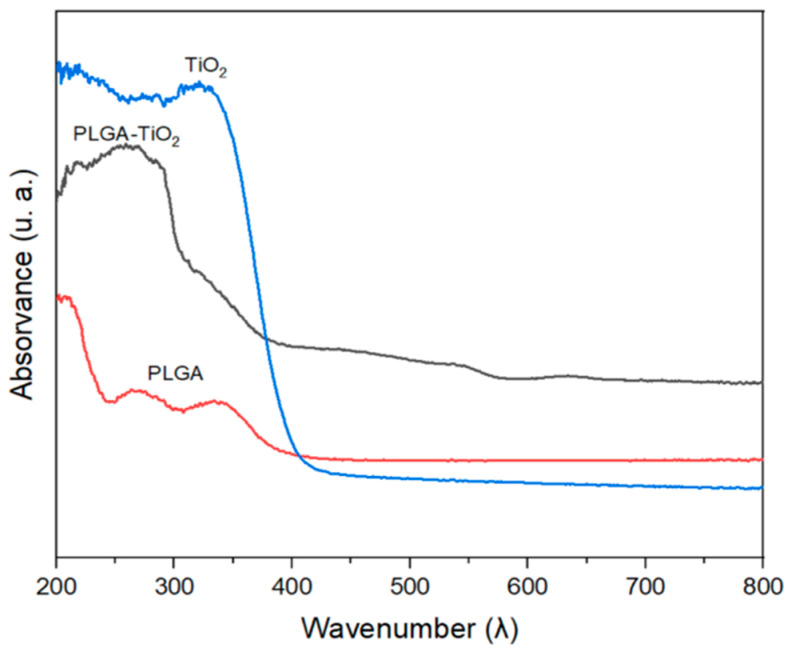
UV–Vis spectra of pure titanium dioxide (TiO_2_), nanocomposite (PLGA–TiO_2_) and poly (lactic-co-glycolic acid) (PLGA).

Figure 3 shows the UV–Vis analysis results of the materials. TiO_2_ reveals good photo-energetic absorption in the range of 200 to 400 nm, indicating that it can be active with natural light radiation [7,16]. The spectrum corresponding to PLGA shows absorption bands at 265 and 338 nm, while the results of the compound PLGA–TiO_2_ absorbs in the range of 200 to 400 nm. However, it does not show the marked bands of TiO_2_ at 324 nm, which may indicate the encapsulation of the nanoparticles [20].

### 2.4. FT-IR

**Figure 4:**
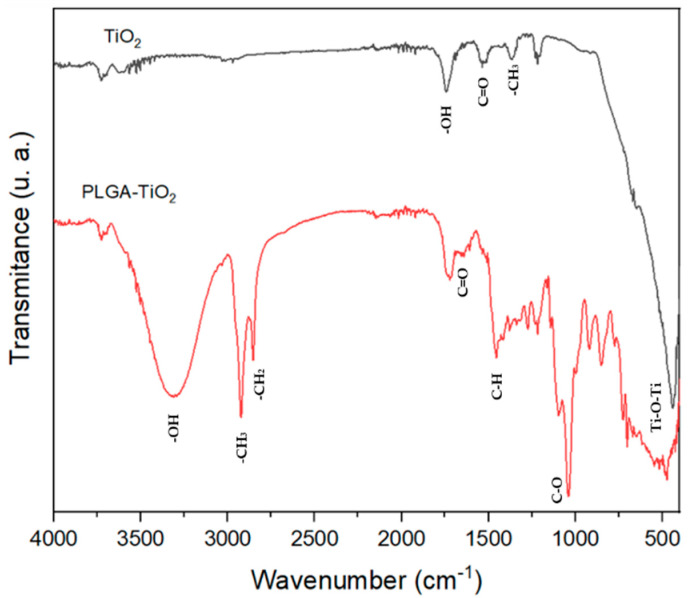
FT-IR spectra of pure titanium dioxide (TiO_2_) and nanocomposite (PLGA–TiO_2_).

Figure 4 shows the molecular vibrations of the TiO_2_ nanoparticles and the PLGA–TiO_2_ nanocomposite. The signals corresponding to TiO_2_ can be observed in the area of 800–400 cm^−1^, and signals corresponding to -OH (1737 cm^−1^) in the spectrum of TiO_2_ and 3305 cm^−1^ in PLGA–TiO_2_ are also observed; this signal is accentuated due to the polymer matrix of the material that encapsulated the extract. In addition, the TiO_2_ spectrum shows signals corresponding to the stretching and bending vibrations of -C=O (1531 cm^−1^) and -CH_3_ (1367 cm^−1^), which agree with what was reported by Serga et al. in 2021 [21]. The PLGA–TiO_2_ spectrum demonstrated absorption bands belonging to C=O ester bonds (1722 cm^−1^) and C-H (1458 cm^−1^) corresponding to a deformation of the O-CH_2_ group of the PLGA [20]. Furthermore, peaks caused by stretching of C-O (1043 cm^−1^), -CH_3_ (2920 cm^−1^), -CH_2_ (2856 cm^−1^) and -OH (3313 cm^−1^) bonds [22] are shown.

### 2.5. Physisorption Analysis

**Figure 5:**
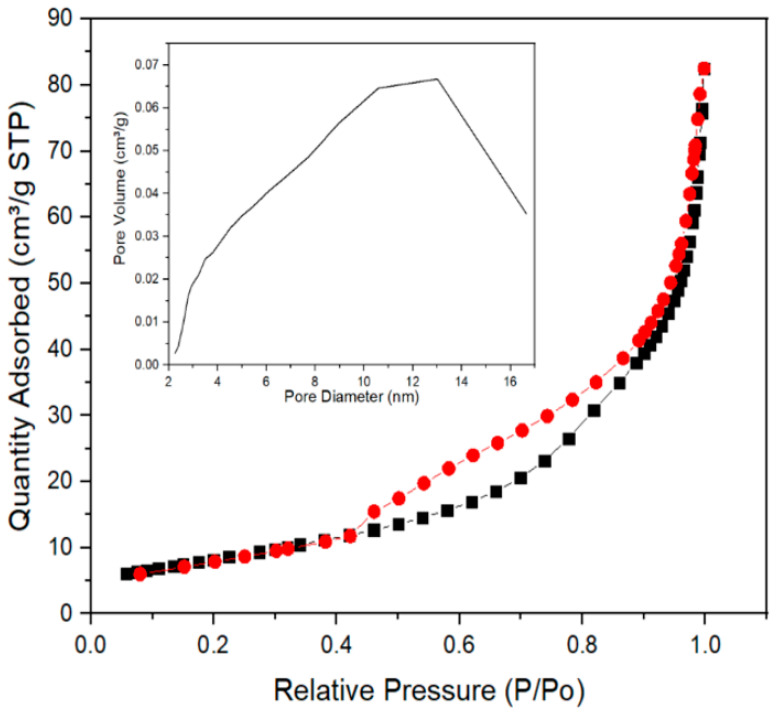
Adsorption–desorption isotherms of N_2_.

Figure 5 shows a type II isotherm, which are macroporous solids where the formation of the monolayer predominates. Characterized by the overlap of the monolayer and multilayer, it presents a type H3 hysteresis in the range of 0.45–0.90 P/P0, indicative of mesoporosity [23]. The Brunauer–Emmett–Teller (BET) method showed a surface area of 1.4864 m^2^/g and the Barrett–Joyner–Halenda (BJH) method a pore size of 5.9109 nm; these determinations allow us to assume that the material has open and oval pores with a non-uniform shape [24] that can act as containers for the extract with which the functionalization was performed.

### 2.6. PLGA–TiO_2_ Release Profile

**Figure 6:**
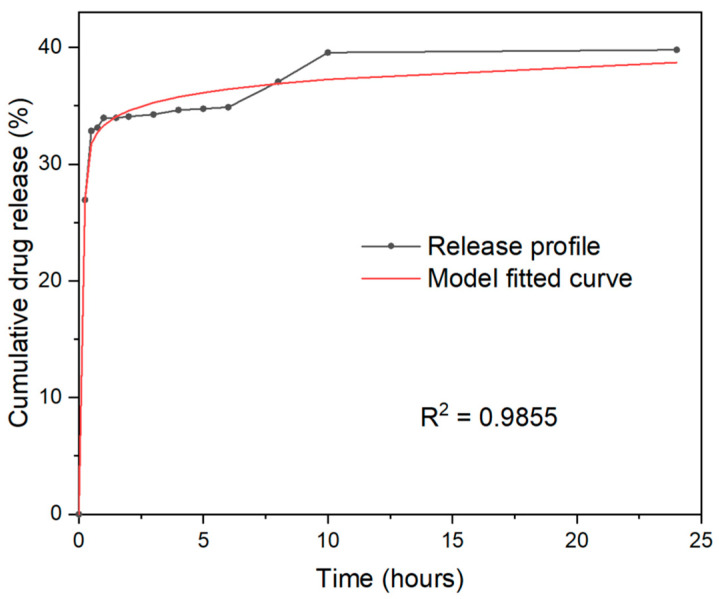
Profile of drug delivery of nanomaterials PLGA–TiO_2_.

The values of drug loading and encapsulation efficiency are summarized in Figure 6. The PLGA–TiO_2_ release profile shows a rapid initial release in the first two hours; previously, the release profile of the PLGA–extract was reported [12]. It can be said that there are significant differences in encapsulation efficiency (EE) and drug loading (DL) between extract–PLGA and TiO_2_–PLGA. These results are consistent with what was reported by Martin-Camacho et al. who observed that as the amount of extract increased, the DL also increased [12]. The release profiles of PLGA loaded with TiO_2_ and extract exhibited a controlled release with a pattern of rapid initial release followed by a sustained release at pH = 7. During the first 15 min, 26.94% of TiO_2_–PLGA was released, and after 90 to 1440 min 39.79% was released. The initial burst is attributed to the weak bonds of the TiO_2_–extract trapped on the surface of the PLGA matrix. While the sustained release is attributed to the diffusion of TiO_2_–extract from the internal matrix of the PLGA. The release profile was analyzed using the add-in program for Microsoft Excel, DDSolver [25]. Based on the analysis, the model in which the release profile fits is the Weibull model with an R squared of 0.9855.

## 3. Materials and Methods

### 3.1. Chemical Reagents

The titanium oxide (TiO_2_) was obtained from titanium butoxide (IV) (C_16_H_36_O_4_Ti, Sigma Aldrich, St. Louis, MO, USA) and a natural extract (international patent No.PCT/IB2020/061916). The functionalization of the material required poly(vinyl alcohol) (PVA) ((C_4_H_6_O_2_)n, Sigma Aldrich, St. Louis, MO, USA) poly(D,L-lactide-co-glycolide) (PLGA) (lactide:glycolide (75:25), Sigma Aldrich, St. Louis, MO, USA) and acetone ((CH_3_)_2_CO, Sigma Aldrich, St. Louis, MO, USA). The releases were made in phosphate-buffered saline (PBS).

### 3.2. Nanomaterial Synthesis

TiO_2_ nanoparticles were synthesized by the sol–gel method [18] with some modifications, using titanium butoxide as a precursor. An amount of 40 mL of butoxide added drop by drop was dissolved in 40 mL of extract in a three-mouth flask. The solution was heated to 80 °C for four hours using magnetic stirring. The solution was then cooled to 0 °C for 18 h. The gel was then dried at 100 °C and calcined at 500 °C for 4 h in a static air atmosphere (heating rate of 2 °C/min).

### 3.3. Nanoparticle Functionalization

The NPs were functionalized following the emulsion solvent evaporation technique, using 5 mL of a solution composed of PVA (4%), extract (16% *w*/*v*), and 5 mg of TiO_2_ NP, sonicated 3 min, and homogenized using an Ultra-turrax (IKA, T18; Germany). An amount of ~5 mg of PLGA (75:25) was added drop by drop and dissolved in 400 μL of acetone. The samples obtained were kept at −80 °C for 2 h and freeze-dried at −50 °C (Labconco, FreeZone 6; Kansas, MO, USA) [12].

### 3.4. Sample Characterization

The morphology of the materials was observed by scanning electron microscopy (MIRA 3LMU, Tescan, London, UK) operated at 20 kV.

The absorption spectra of the materials were acquired by a UV–Vis DRS (Shimadzu UV-2600, Tokyo, Japan) provided with an integration sphere suitable for diffuse reflectance studies. The UV–Vis DRS spectra were obtained from 190 to 900 nm wavelength.

The X-ray powder diffraction patterns were acquired using an XRD Panalytical diffractometer (Empyrean, Almelo, The Netherland) equipped with Cu Kα radiation (𝜆 = 0.154 nm). Data were collected from 10° to 90° (2*θ* with a scan rate of 0.02°/0.2 s. The average crystal size was determined using the Scherrer Equation (1):

(1) $$D=\frac{k\lambda }{\beta \cos\theta }$$

where *D* is the crystal size, *k* is the form factor (0.89), *λ* is the wavelength of Cu Kλ radiation, β is the width evaluated at mid-high of the most intense diffraction peak, and *θ* is the Bragg angle. The inter-planar distance (d) can also be evaluated from Bragg’s law (2):

(2) 2dsinθ=nλ

The FT-IR spectra for the material was recorded with an FTIR (Shimadzu, IRTracer-100, Tokyo, Japan) spectrophotometer using attenuated total reflectance (ATR) with a diamond waveguide (XR model). A detector of fast recovery deuterated triglycine sulfate (DTGS) (standard) was used for the analysis. The spectra were recorded at room temperature, with 24 scans and 4 cm^−1^ of resolution and from 4000 cm^−1^ to 400 cm^−1^. The equipment measures interferogram signals that must be decoded. For this, a mathematical technique called Fourier transform (FT) is used, which generates the change of the interferogram signals to the frequency domain; this process is carried out by the computer of the equipment and at the end, it presents the user with the spectral information obtained from the analysis [26].

A micromeritics TriStar II Plus (Norcross, GA, USA) was used to determine the specific surface area of adsorption–desorption isotherms of N_2_ at 77 K. The BET and BJH methods were used to calculate the specific surface area and mean diameter of pore, respectively.

### 3.5. Release Profile of the Extract in PLGA-TiO_2_

The dialysis method was used, suspending 5.0 mg of NP in 5 mL of buffer at different pH (1.5 and 7.0) to simulate physiological conditions. The suspension was maintained at 37 °C and 150 rpm. Samples were taken at 15, 30, 45, 60, 90, 120, 180, 240, 300, 360, 420, 480, 540, 600, and 1440 min and read on a UV–Vis spectrophotometer (Shimadzu UV-2600, Tokyo, Japan) at 256 nm. Release kinetics were adjusted to kinetic models to determine reaction order and release mechanism [13].

### 3.6. Evaluation of Extract Encapsulation Efficiency

For the evaluation of the extract encapsulation efficiency (EE%) and drug load (DL%), 5 mg of functionalized nanoparticles were placed in 5 mL of buffer phosphates and the solution was sonicated for 10 min. Subsequently, it was stirred at 37 °C for 48 h. Finally, the solution was centrifuged for 15 min at 3000 rpm, using the supernatant for reading on a UV–Vis spectrophotometer (Shimadzu UV-2600, Tokyo, Japan) at 276 nm [19]. Drug load and encapsulation efficiency were determined by the following equations:

$$EE\%\;=\frac{\;free\;drug\;mass}{drug\;used\;for\;synthesis}\;\times 100\%$$

$$DL\%\;=\;\frac{free\;drug\;mass}{nanoparticles\;mass}\;\times 100\%$$

## 4. Conclusions

The XRD, FTIR, and UV–Vis analyses showed that the green synthesis of the nanocomposite (PLGA–TiO_2_) does not modify the crystallinity of the material, maintaining the anatase phase, in addition to confirming the inclusion of TiO_2_ nanoparticles within the PLGA matrix. SEM, BJH, and BET analyses determined that the material has a spherical morphology with a surface area of 1.4864 m²/g and an approximate pore size of 5.9 nm. These results demonstrate the potential of the PLGA–TiO_2_ nanocomposite for possible pharmaceutical and nanobiomedical applications due to its stable release. Defining the final applicability of the nanopolymer studied requires tests such as cell viability, cytotoxicity, and antibacterial tests that are recommended for future research.

## Figures and Tables

**Table 1:** Comparison of the crystallinity parameters of titanium dioxide.

Material	Lattice Parameters		Crystallite Size (nm)
	a = b (Å)	c (Å)	
TiO_2_	3.774 ^a^	9.426 ^a^	11.97 ^b^
TiO_2_ reported [19]	3.784	9.478	21.6

^a^ Calculated by Bragg’s law. ^b^ Obtained by Scherrer equation.

## Data Availability

Not applicable.
